# Methodology for evaluating Insite: Canada's first medically supervised safer injection facility for injection drug users

**DOI:** 10.1186/1477-7517-1-9

**Published:** 2004-11-09

**Authors:** Evan Wood, Thomas Kerr, Elisa Lloyd-Smith, Chris Buchner, David C Marsh, Julio SG Montaner, Mark W Tyndall

**Affiliations:** 1 British Columbia Centre for Excellence in HIV/AIDS, St. Paul's Hospital; Vancouver, BC, Canada; 2 Department of Medicine; Faculty of Medicine; University of British Columbia; Vancouver, BC, Canada; 3 Canadian HIV/AIDS Legal Network; Canada; 4 Vancouver Coastal Health; Vancouver, BC, Canada

## Abstract

Many Canadian cities are experiencing ongoing infectious disease and overdose epidemics among injection drug users (IDUs). In particular, Human Immunodeficiency Virus (HIV) and hepatitis C Virus (HCV) have become endemic in many settings and bacterial and viral infections, such as endocarditis and cellulitis, have become extremely common among this population. In an effort to reduce these public health concerns and the public order problems associated with public injection drug use, in September 2003, Vancouver, Canada opened a pilot medically supervised safer injecting facility (SIF), where IDUs can inject pre-obtained illicit drugs under the supervision of medical staff. The SIF was granted a legal exemption to operate on the condition that its impacts be rigorously evaluated. In order to ensure that the evaluation is appropriately open to scrutiny among the public health community, the present article was prepared to outline the methodology for evaluating the SIF and report on some preliminary observations. The evaluation is primarily structured around a prospective cohort of SIF users, that will examine risk behavior, blood-borne infection transmission, overdose, and health service use. These analyses will be augmented with process data from within the SIF, as well as survey's of local residents and qualitative interviews with users, staff, and key stakeholders, and standardised evaluations of public order changes. Preliminary observations suggest that the site has been successful in attracting IDUs into its programs and in turn helped to reduce public drug use. However, each of the indicators described above is the subject of a rigorous scientific evaluation that is attempting to quantify the overall impacts of the site and identify both benefits and potentially harmful consequences and it will take several years before the SIF's impacts can be appropriately examined.

## Introduction

Many Canadian cities are currently experiencing Human Immunodeficiency Virus (HIV) and hepatitis C virus (HCV) epidemics as a result of illicit injection drug use [1,2]. Other costly infectious diseases that can be easily acquired from non-hygenic injection practices, such as endocarditis and cellulitis, are also common [3]. The health of injection drug users (IDUs) is further compromised by avoidance and erratic use of primary care services, costly emergency room visits, and acute care hospitalizations [3-6]. Public drug use also occurs in many inner city neighborhoods, and public drug use and the unsafe disposal of syringes is a major community concern [7,8].

In over two dozen European cities and more recently in Sydney, Australia, safer injection facilities (SIFs), where injection drug users can inject pre-obtained illicit drugs, have been implemented in an effort to reduce the community and public health impacts of illicit drug use [9]. SIF typically have several primary objectives including: the reduction of public drug use, fatal and non-fatal overdose, and infectious disease risk; improving contact between a highly marginalized 'at-risk' population and the healthcare system; and enhancing recruitment into medical care and addiction treatment [9-11]. Within SIFs, IDUs are provided with clean injecting equipment, medical attention in the event of overdose, as well as access to or referral to primary healthcare and other services including addiction treatment.

While it must be stressed that limited quantitative data are presently available, various reports have credited SIFs with a number of public health and community benefits including: improving the health and social functioning of their clients [11], while reducing overdose deaths [12], risk behaviors known to transmit infectious diseases [13], improperly discarded syringes [14], and public drug use [15]. In addition, improved access to medical care and drug treatment has been attributed to SIF attendance [10,16]. A limitation of these earlier analyses is that, in a number of settings, there has not been a commitment on the part of health agencies to fund comprehensive evaluations, and in many instances there have not existed prospective cohorts to inform examinations of SIF's impacts [17].

On September 22, 2003 Vancouver, Canada opened North America's first government sanctioned SIF pilot study [18]. Federal government approval for the three-year pilot study was granted on the condition that the health and social impacts of the SIF be the subject of a rigorous scientific evaluation. More recently, several Canadian cities have begun to consider their own SIF evaluations, including Montreal and Victoria [19,20]. Since several years were devoted to the development of the Vancouver SIF evaluation methodology, and since the investigators wished to be as open with methodology as possible [21], the present article was prepared to describe the framework of the evaluation and to report on preliminary observations. The publication of these observations may also be useful for other Canadian considering initiating SIF trials [19,20].

### Client Anonymity

Prior to the opening of the SIF, a major concern with the evaluation related to willingness of the target community to use the injection facility [18]. In order to attract the target population without raising fears about confidentiality, and to make the service as low threshold as possible, all clients of the SIF can remain anonymous. Since fears regarding reduced willingness to use SIF, if client registration was required, were observed in feasibility studies conducted prior to Insite's opening [18], the SIF operated as a completely low threshold service in the first 6 months of operation and maximizing access to the SIF was the top priority. During this time only paper records were maintained. After 6 months of operation, and after trust was developed between the SIF operators and the target community, service use was tracked at an individual level using a database that tracks all client service use and outcomes within Insite. The phasing in of a digital tracking system was successful, although service uptake was so substantial and immediate after the site opened, it is not known if this was necessary. A further challenge was the ethical dilemma posed by providing a health service that must also be rigorously evaluated [22]. Specifically, it was apparent to the investigators that it would be unethical to limit use of the SIF to those who agreed to participate in research. Instead, equipoise was reached by allowing participation in surveys and other aspects of the research to be optional to SIF users.

### Aims of Insite

In brief, the aims of Insite are to reduce public injection drug use and the unsafe disposal of syringes in public spaces, the reduction of overdoses and infectious disease risk, and improve access to healthcare services among IDUs. The methodology for evaluating these aims is described below and involves both a prospective cohort design and additional data sources including evaluation of community impacts.

## Evaluation Methodology

### Data Sources

The framework for the Vancouver SIF evaluation was designed prior to the SIF's opening and involved a number of methodological approaches. In light of the lack of existing quantitative efficacy data [17], the existence of ethical concerns [22], and an awareness that a non-randomized studies may be vulnerable to substantial selection biases [23], the Vancouver SIF evaluation is primarily structured around a prospective cohort design that involves the longitudinal measurement of a number of outcomes including blood-borne infection and overdose incidence, risk behavior, drug use practices, such as public drug use, and health services use.

The Vancouver SIF evaluation is somewhat unique because of the availability of a number of pre-existing data sources. These data sources include the community health and safety evaluation (CHASE) cohort, which is a community recruited virtual cohort of Downtown Eastside residents that prospectively and retrospectively examines health service use in the community by linking to administrative health record databases. In addition, the Vancouver Injection Drug Users Study (VIDUS) is an ongoing prospective cohort study of injection drug users that involves semi-annual serology of HIV and HCV as well as a semi-annual questionnaire [24]. VIDUS and CHASE allow for the description of IDUs in the community who are using Insite and a comparison between those that are and are not using the service.

In addition, in order to augment these data sources and to allow for close examination of the characteristics of Insite clients over time, a prospective cohort of Insite users has also been established. The Scientific Evaluation of Supervised Injecting (SEOSI) cohort is based on a representative sample of Insite users. The sample is derived through random recruitment of Insite users who are offered an informed consent to enroll into the study. Random recruitment involves attending the SIF at times of the day that are randomly selected using a random number generation program in SPSS, and inviting all users who use the SIF at this time to enroll in the study. As with VIDUS, participants provide a blood sample and conduct an interviewer-administered questionnaire. The SEOSI questionnaire deals with items that are particularly relevant to Insite, such as risk behaviours, public drug use, satisfaction with Insite, and access to medical care and addiction treatment services. All SEOSI participants provide informed consent to link to the Insite database so that SIF use can be tracked, as well as informed consent to access administrative health record databases in the community. As of September 1, 2004 over 900 Insite users have been enrolled into SEOSI and comparisons of socio-demographic variables (age, gender, etc) has shown that the SEOSI cohort is statistically similar to the overall cohort of insight users (all *p *> 0.05).

### Client Satisfaction

Measures of client satisfaction are compiled as part of the SEOSI questionnaire. Through ratings of service quality in terms of the 5 SERVQUAL dimensions: Tangibles (e.g., the appearance of the physical facilities); Reliability (e.g., the ability of staff to perform the service dependably); Responsiveness (e.g., the willingness of staff to help clients and provide prompt service); Assurance (e.g., security, credibility and courtesy); and Empathy (e.g., ease of access, approachability and effort taken to understand clients' requirements). Similarly, reasons for avoiding the service are measured among IDUs in VIDUS who have not used Insite.

### Additional Data Sources

These above prospective cohort data will be augmented by a number of other data sources including: process indicators, measures of community satisfaction and perceived impact, standardized measures of public order, and qualitative and quantitative measures of the health of the target population. The collection of each of these data sources is described below.

### Process Measures

In order to track service use in the database at an individual level, while allowing for participant anonymity, each client must select a unique client 'handle' or nickname. The SIF database has a search function that allows for rapid searches based on demographic information, such as birth date, if an individual forgets their handle. Similar anonymous tracking of individual clients is commonly used at needle exchanges and other services for illicit injection drug users [25].

A primary purpose of the evaluation is to measure process indicators related to service uptake within the SIF, and this is enabled through the Insite database. The database tracks what drugs participants are consuming (heroin, cocaine, etc) and what services, such as nursing care and counseling services, are accessed by each client. For instance, in the month of May 2004, over 1300 unique visits were logged into the database.

### Community and Staff Satisfaction

Community satisfaction and the perceived impact of the SIF on business persons are measured through a community survey that is performed in person among street recruited residents and at street-level businesses. The survey is similar to surveys being used in the Sydney SIF trial, and examines perceived changes in the neighborhood after the SIF's opening. In addition, staff satisfaction with the operation of the facility is measured through focus groups and qualitative interviews with staff persons. These interviews focus on how service delivery can be improved and on what measures can be taken to ensure staff safety and satisfaction.

### Public Order

Standardized measures of public order were undertaken to examine the impact of the SIF on several indicators of public injection drug use. In brief, the survey protocol involves measuring specified public order indicators within an a priori defined geographical area in the neighborhood and at a priori defined times of the week. Data collection times are spread evenly throughout the week and involved walking through the study zone in the same pattern. Measures of discarded syringes, injection-related litter, and public injection drug use are all measured prospectively. An evaluation of these indicators has recently been described in detail [26].

## Preliminary observations

Following the opening of the SIF in September 2003, there was widespread support among the target population with a steady increase in uptake during the first few weeks. The site reached virtual capacity within two months and currently an approximate average of 500 injections take place each day in the site. The busiest times of the day are mid-afternoon and early evening at which times demand often exceeds capacity and waiting times to get into the 12 seat injection room can result in participants obtaining syringes and injecting elsewhere. Whether the wait times are disproportionately affecting specific populations is presently being investigated. Utilization also fluctuates daily, peaking on the days leading up to, and following welfare day. Exit surveys of IDU clients have been widely supportive of the service and high levels of satisfaction with the service among Insite staff have been reported. Contrary to the suggestion that cocaine users would be unwilling to use the SIF [9], approximately half of all injections include cocaine.

Despite the chaotic behaviours often associated with injection drug use, overall staff safety has been high and the instances of verbal or physical abuse by clients are managed efficiently as per the service's protocols. In outstanding circumstances, Vancouver Police Department has been called to remove disruptive clients, and support and assistance from the police in this regard has been very positive. Overall the staff remains very committed to the activities at Insite and staff satisfaction has been high.

Overdoses, from a range of illicit drugs, are commonly observed in the SIF. The severity of these overdoses range from lowered respiration rate to severe emergency situations that have required the administration of naloxone and ambulance responses. Given the high levels of illness (for instance HIV and hepatitis C co-infection) and drug using behaviours (unknown substances of unknown purity) of the target population, it is not inconceivable that a fatality could occur in the SIF despite staff supervision and emergency response.

There have been no instances where used syringe borrowing has been seen within Insite. These behaviours are common among street based injectors and it is well recognized that these activities promote the spread of blood-borne infections. It is also noteworthy that alcohol swabs to clean the injection site, and clean water and cookers are all provided to optimize hygenic injection procedures. Research of street-based IDU in Vancouver has shown that alcohol swabs are rarely used, and that non-hygenic water sources, such as puddle water, are commonly used. It is also noteworthy that within the SIF, safer hygenic injection practices are taught by the nursing staff to IDUs who have never been shown how to inject safely.

In addition to supervising injections, teaching safer injecting practices, and responding to overdoses, there has been substantial health intervention within Insite. In particular, referrals to medical care at St Paul's Hospital are common as well as referrals to community health centres. Early intervention for primary medical care concerns, such as abscesses, is commonly provided by the Insite nursing team, and coverage with public health interventions, such as flu shots, has been provided to Insite users. In addition, addictions counseling occurs on site and there have been many referrals to detoxification programs and methadone maintenance therapy.

## Summary

Overall, Insite has attracted the target population and preliminary evidence suggests that the experiences within Insite as well as the community impact have been consistent with the experience of over two dozen European settings where SIF exist, and more recently Sydney, Australia. The examination of early changes in public order has been completed and there is strong evidence of improvement in several indicators including public drug use [26].

However, each of the indicators described above is the subject of a rigorous scientific evaluation that is attempting to quantify the overall impacts of the site and identify both benefits and potentially harmful consequences over a multi-year period. This evaluation is primarily structured around a prospective cohort design that will involve the longitudinal measurement of health and community indicators over the next several years. As such, it will be some time before the overall impact of Insite on a number of outcomes, such as blood-borne infections and IDUs behavior, can be adequately quantified.
